# Pregnancy with Takayasu’s Arteritis: A Case Report and Literature Review

**DOI:** 10.7759/cureus.3370

**Published:** 2018-09-26

**Authors:** Rayan Itani, Naela Elmallahi, Mohamed Ahmed Abdelmoneam Ramadan, Abdullah Al Ibrahim

**Affiliations:** 1 Obstetrics and Gynecology, Women's Wellness and Research Center/ Hamad Medical Corporation, Doha, QAT; 2 Obstetrics and Gynecology, Women's Wellness and Research Center/ Hamad Medical Corporation, Doha , QAT

**Keywords:** takayasu's arteritis, pregnancy, women hospital, qatar

## Abstract

Takayasu's arteritis (TA) is a rare and chronic inflammatory disease of the large vessels. It affects women of reproductive age and leads to an increased risk of cardiovascular complications, such as hypertension and congestive heart failure. We are presenting a case of a pregnant woman with TA, who was seen and managed at a tertiary care institute and ultimately enjoyed a favorable outcome. Thus, multidisciplinary care for patients with TA has proven to be crucial in optimized and favorable maternal and fetal/neonatal outcomes.

## Introduction

Takayasu's arteritis (TA), also known as “young female arteritis,” is a rare and chronic inflammatory disease of the large vessels. The disease mainly affects women of reproductive age and Asian origin [1]. Moreover, TA leads to several complications including occlusion as well as aneurysm formation in systemic and pulmonary arteries. During pregnancy, there is an increased risk of cardiovascular complications such as hypertension and congestive heart failure [2]. However, evidence remains scarce on the appropriate management of the disease despite its harmful impact on maternal and neonatal health. Moreover, the literature supports the need for an interdisciplinary approach to optimize the care of such patients [3].

## Case presentation

**Figure 1:**
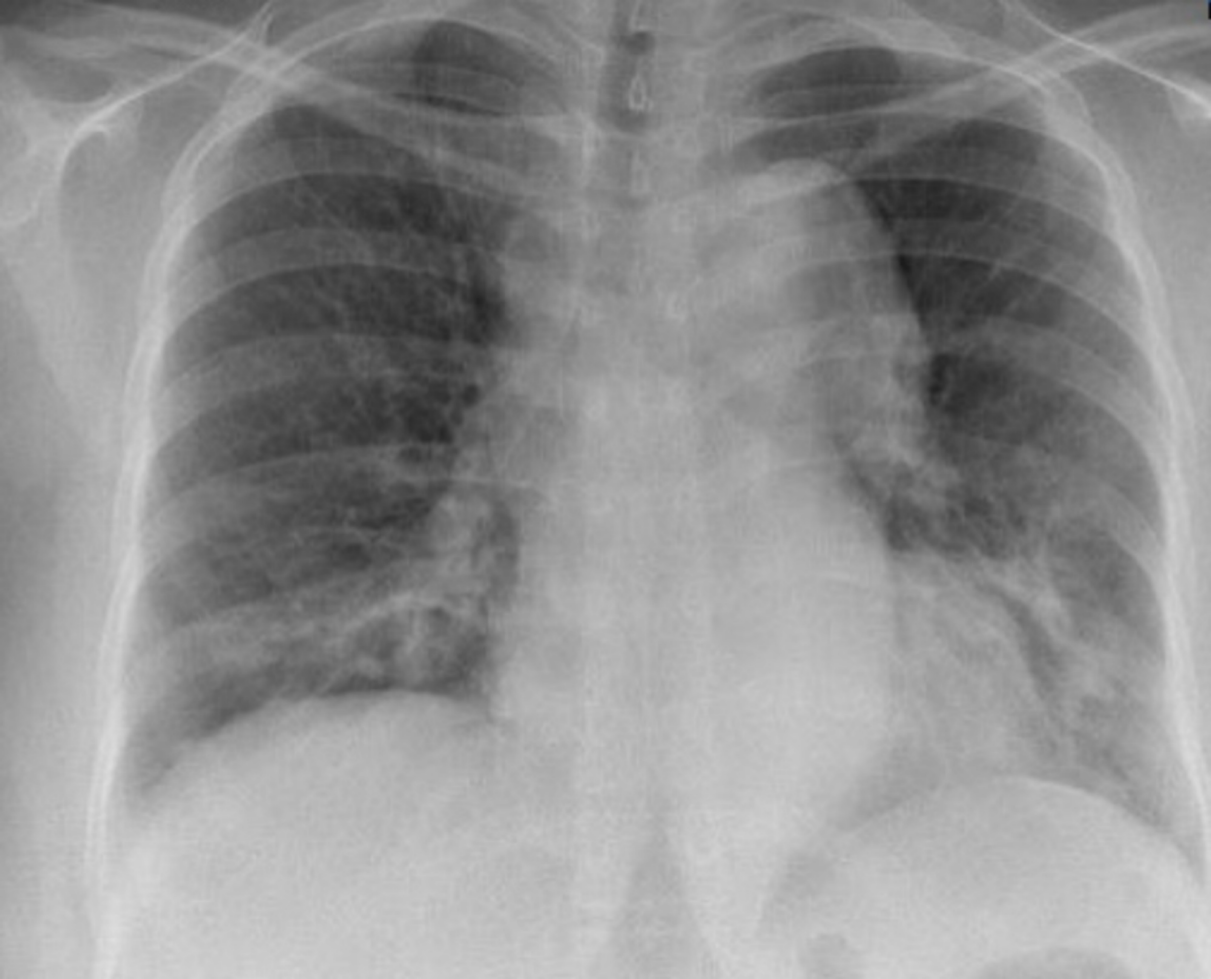
X-ray showing widened mediastinum, 2014.

A 31-year-old woman, of Indian origin and known to have TA, was booked for an antenatal care appointment at the 11th week of her third gestation in April 2017. Her earlier two pregnancies in 2008 and 2010 resulted in the normal vaginal delivery of a full term and healthy singleton each. However, her second pregnancy was complicated by pre-eclampsia. The patient was incidentally diagnosed through a routine chest X-ray, which revealed a widened superior mediastinum (Figure 1) in June 2014.

**Figure 2:**
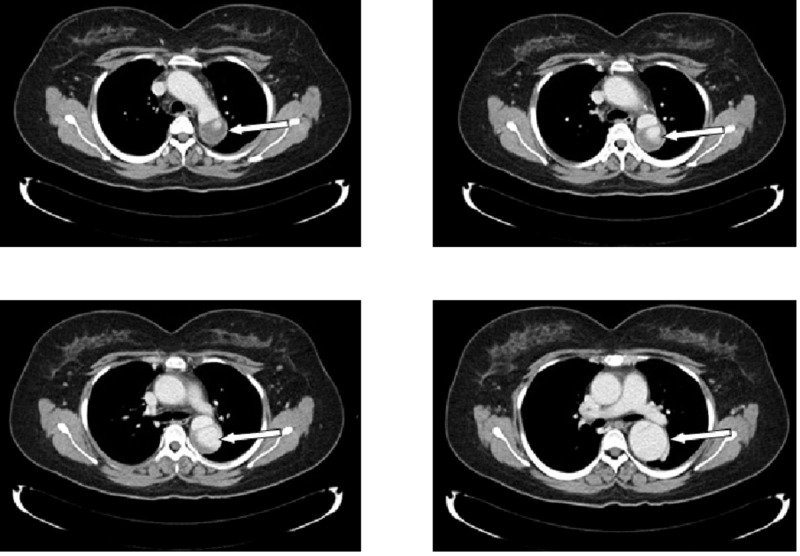
Computed tomography (CT) scan showing a dissecting aortic aneurysm (white arrow), 2014.

Further physical examination revealed an absent left radial pulse. Subsequently, a computed tomography (CT) pulmonary angiogram confirmed the presence of a dissecting aortic aneurysm, which measured 7 cm in length and was inferior to the origin of the left subclavian artery (occluded) (Figure 2). The CT scan also revealed an atrophic right kidney and a hypertrophied left one. Thus, the patient was placed on prednisone as well as methotrexate and booked for endovascular repair surgery. The woman later successfully underwent endovascular stent grafting of the thoracic aorta in April 2015. After developing pneumonia, the patient was switched from methotrexate to azathioprine (AZA) and maintained on amlodipine (5 mg), aspirin, and prednisone.

The lady's antenatal care was under the feto-maternal unit (FMU) at the Women’s Hospital. Simultaneously, the patient was regularly followed by her rheumatologist and vascular surgeon. The patient resumed her medication during pregnancy; and serial ultrasound scans revealed a normally developing fetus. The antenatal period was uneventful except for gestational diabetes mellitus. In November 2017, the patient presented at 38+ weeks gestation for an elective cesarean section and bilateral tubal ligation as advised by the multidisciplinary team. Thus, the patient delivered a healthy baby girl, weighing 2279 g, and her postpartum period was uneventful. The patient was counselled about breastfeeding while on AZA and told that the current evidence does not suggest any risk from AZA during pregnancy and while breastfeeding. One month following her delivery, the patient presented for a follow‐up appointment with her vascular surgeon and was asymptomatic. Also, her blood pressure (BP) measurement and inflammatory markers were within the normal range.

## Discussion

Women account for 80%-90% of TA cases and the age of onset is usually between 10 and 40 years [4-5]. The disease has a worldwide distribution, with the greatest prevalence being in Asia [6]. Furthermore, Japan reported an estimated 150 new cases of TA each year [7]. On the other hand, the incidence of TA in the United States and Europe is one to three new cases per year per million population [5].

Takayasu's arteritis is a chronic inflammatory disease that involves the aorta, its branches, and the pulmonary arteries [6, 8]. The inflammation results in varying degree of stenosis, occlusion, or dilatation of the involved vessels. The etiology and the precise pathogenesis of TA are still unknown; however, much has been learnt about the disease since its initial description by M. Takayasu, a Japanese ophthalmologist, in 1908 [9-10]. Moreover, the symptoms range from fever, fatigue, and weight loss to life-threatening hemoptysis and heart failure. The management of TA is a multidisciplinary approach with the involvement of obstetricians, anesthesiologists, cardiologists, rheumatologists, and neonatologists. Ultimately, the aims encompass the control of inflammation, prevention, and treatment of complications like hypertension and occlusive or stenotic lesions [11].

When managing women of reproductive age with TA, preconception counseling is essential. In addition, such counseling will focus mainly on dosage adjustment, cessation of cytotoxic drugs, folic acid supplementation in the periconception period, and the optimal timing of pregnancy. Similarly, the pregnancy should be ideally planned in remission phase and patients are encouraged to pursue an early booking for regular antenatal supervision. In addition to routine antenatal visits, serial monitoring of BP, renal function, cardiac status, and pre-eclamptic screening is vital in such patients. Furthermore, fetal surveillance is also necessary and will include daily fetal kick count, gravidogram, serial fetal biometry, biophysical profile, and fetal Doppler [12].

In addition to that, pregnancy does not interfere with the disease progression of TA [13-14]. However, BP control is of paramount importance as any increase might rupture an aneurysm, induce hypotension, and lead to cerebral ischemia in the mother. Moreover, peripheral BP monitoring may not be accurate and might complicate the treatment of hypertension in these patients. Most patients benefit from invasive BP monitoring or the measurement of peripheral BP in multiple extremities because the upper extremity pulses may be absent [15].

Also, controlling BP during pregnancy may be difficult due to the physiological changes in this period. Thus, any patient with TA should plan to conceive when the BP and disease are stable. It is also vital to adjust the antihypertensive medication and avoid angiotensin-converting enzyme inhibitors or angiotensin inhibitors. On the other hand, uncontrolled hypertension during pregnancy has been associated with abortion, stillbirths, aortic dissection, cardiac and renal insufficiency, stroke, and maternal death [16-18]. Finally, vaginal delivery has proven to be the preferred mode of labor management for patients with TA. Additionally, epidural analgesia has been advocated for labor and delivery as well.

## Conclusions

The current case adds further evidence to the medical management of TA during pregnancy. As reported earlier, the incidence of TA is the highest during childbearing age, but there appears to be no exacerbating effect of pregnancy on the natural history of TA. Multidisciplinary care for patients with TA has proven crucial to reach optimized and favorable maternal and fetal/neonatal outcomes.
